# Vulnerability of anterior medial temporal lobe subregions to early tau‐related neurodegeneration in Alzheimer's disease: Converging evidence from tau‐PET and plasma p‐tau217

**DOI:** 10.1002/alz.71571

**Published:** 2026-06-17

**Authors:** Nidhi S. Mundada, Niyousha Sadeghpour, Emily McGrew, Hannah L. Tucker, Ilya M Nasrallah, Sandhitsu R. Das, David A. Wolk, Paul A. Yushkevich, Christopher A Brown, Laura E. M. Wisse

**Affiliations:** ^1^ Department of Bioengineering University of Pennsylvania Philadelphia Pennsylvania USA; ^2^ Department of Radiology Perelman School of Medicine at the University of Pennsylvania Philadelphia Pennsylvania USA; ^3^ Department of Neurology Perelman School of Medicine at the University of Pennsylvania Philadelphia Pennsylvania USA; ^4^ Department of Clinical Sciences Lund Lund University Lund Sweden

**Keywords:** Brodmann area 35, medial temporal lobe, neurodegeneration, plasma phosphorylated tau 217 (p‐tau217), preclinical Alzheimer's disease, structural magnetic resonance imaging, tau pathology, tau positron emission tomography (PET)

## Abstract

**INTRODUCTION:**

The anterior medial temporal lobe (MTL), including the entorhinal cortex (ERC) and Brodmann area 35 (BA35), is among the earliest cortical sites of tau pathology in Alzheimer's disease (AD), yet conventional image segmentation methods poorly capture these regions.

**METHODS:**

We applied an automated segmentation approach using an extended Automatic Segmentation of Hippocampal Subfields (ASHS) atlas, including anterior MTL subregions, in 448 Pennsylvania Alzheimer's Disease Research Center participants with magnetic resonance imaging, tau positron emission tomography (PET) (*n* = 199), and/or plasma phosphorylated tau 217 (p‐tau217) (*n* = 377). Amyloid beta (Aβ) positivity was defined using PET or plasma.

**RESULTS:**

Tau‐PET showed an anterior–posterior gradient, with highest uptake in BA35, ERC, and anterior hippocampus. Increased MTL tau‐PET uptake and plasma p‐tau217 were associated with cortical thinning localized to BA35 and ERC, even in cognitively unimpaired Aβ‐positive individuals.

**CONCLUSIONS:**

Anterior MTL subregions, especially BA35, show early vulnerability to tau‐related neurodegeneration. Extended anterior MTL parcellation improves localization of early tau‐associated structural changes and may facilitate biological staging in preclinical AD.

## Highlights

- Refined anterior MTL imaging sensitively detects early tau‐related neurodegeneration.
- An anterior‐to‐posterior gradient of tau pathology was revealed in vivo.
- Anterior MTL structural changes track closely with tau‐PET and plasma p‐tau217.
- Early tau‐related neurodegeneration localizes to ERC and BA35.

## 1 INTRODUCTION

Alzheimer's disease (AD) is defined by the accumulation of extracellular amyloid beta (Aβ) plaques and intracellular tau neurofibrillary tangles (NFTs), with tau pathology being especially important for understanding the clinical expression of disease. In contrast to Aβ, tau typically spreads in a stereotyped pattern across cortical regions typically beginning in the anterior medial temporal lobe (MTL), particularly within Brodmann area 35 (BA35; also called transentorhinal cortex) and the entorhinal cortex (ERC), before extending into the hippocampus and amygdala and, ultimately, neocortical association areas.^1^, ^2^, ^3^, ^4^ Recent advances in tau positron emission tomography (PET) have enabled in vivo assessment of tau pathology, with studies demonstrating early tracer uptake in MTL regions, particularly the ERC, among some Aβ‐positive (Aβ+) individuals with or without symptoms.^5^, ^6^ More recently, plasma assays for phosphorylated tau, particularly phosphorylated tau 217 (p‐tau217), have emerged as scalable measures of AD‐related tau pathology^7^, ^8^ that correlate strongly with concurrent tau‐PET burden and similarly predicts cognitive decline.^9^

Autopsy studies have consistently shown that NFT burden correlates strongly with neuronal loss and local atrophy, particularly in the MTL.^10^, ^11^ Imaging studies extend this evidence, demonstrating that higher tau burden is associated with cortical thinning, hippocampal atrophy, and steeper trajectories of cognitive decline.^12^, ^13^, ^14^, ^15^, ^16^, ^17^, ^18^, ^19^ Despite this biological relevance, structural magnetic resonance imaging (MRI) markers in the field have traditionally centered on hippocampal volume, which, while well validated in both clinical^20^ and trial settings,^21^ provides only a coarse summary of disease‐related neurodegeneration.

Growing evidence indicates that anterior MTL subregions may provide earlier markers of AD‐related neurodegeneration. For example, thinning of ERC has been observed in cognitively unimpaired (CU) older adults who later progress to mild cognitive impairment (MCI) or dementia.^22^ However, conventional structural imaging pipelines face three key limitations when applied to the anterior MTL: (1) reliance on coarse anatomical parcellations that do not subdivide the hippocampus or MTL cortex in more granular regions, such as BA35 and anterior hippocampus, which are meaningful for early AD, (2) use of single‐atlas approaches that fail to capture substantial inter‐individual variability in MTL anatomy, making them prone to mislabeling,^23^ and (3) partial inclusion of dura in ERC segmentations, reducing the reliability of thickness estimates.^24^ Collectively, these limitations constrain the sensitivity of standard MRI measures for detecting early, focal neurodegeneration within the anterior MTL.

The Automatic Segmentation of Hippocampal Subfields‐T1 (ASHS‐T1) pipeline was developed to address these challenges.^23^, ^24^ Using a multi‐atlas label fusion strategy, ASHS integrates information across multiple expert‐labeled atlases to improve anatomical accuracy and account for individual variability. The method enables fine‐grained segmentation of MTL subregions, including anterior and posterior hippocampus, ERC, and BA35, and was recently extended to cover the amygdala and anterior parts of MTL cortical areas,^25^, ^26^ as well as surface‐based vertex‐wise thickness mapping, allowing spatially resolved analyses across thin cortical ribbons such as BA35 and ERC.^22^ These innovations make it possible to characterize subtle, anatomically precise structural changes in the anterior MTL territories where tau pathology first emerges. While prior studies have examined MTL subregions using earlier segmentation frameworks, these approaches did not fully capture anterior MTL territories or explicitly differentiate key regions such as BA35 (≈transentorhinal cortex), limiting sensitivity to the earliest sites of tau‐related neurodegeneration.

Leveraging these advances, we examined whether structural changes in anterior MTL subregions, measured using the extended ASHS‐T1 pipeline,^25^, ^26^ were associated with tau pathology measured by tau‐PET and plasma p‐tau217. We evaluated cognitively unimpaired and impaired individuals across the AD continuum (defined by Aβ+), with particular attention to CU Aβ+ participants to capture preclinical effects. We examined tau‐PET uptake on MTL surface maps to assess correspondence with established neuropathological staging and test whether greater tau burden was associated with localized neurodegeneration within anterior MTL subregions. By leveraging complementary Core 2 PET and Core 1 biofluid biomarkers, our goal was to establish convergent evidence that structural imaging of anterior MTL subregions captured tau‐related neurodegeneration. Such findings would support anterior MTL imaging measures as promising biomarkers for the early detection of neurodegeneration, staging, and therapeutic monitoring in AD.

**RESEARCH IN CONTEXT**

1. **Systematic review**: We reviewed literature using PubMed and recent conference proceedings focused on MTL tau pathology and imaging in AD. Prior neuropathological and in vivo studies highlighted early tau accumulation within the ERC and BA35, but conventional MRI approaches are too coarse to quantify these granular anterior MTL regions accurately.
2. **Interpretation**: Our findings demonstrate that using a specialized, anatomically precise MTL segmentation approach enables fine‐grained assessment of anterior MTL subregions, revealing convergent tau‐PET and plasma p‐tau217 associations with cortical thinning localized to ERC and BA35. These results provide in vivo support for the selective vulnerability of anterior MTL regions in preclinical and early AD.
3. **Future directions**: Refining anatomical precision in MTL imaging may improve detection of early neurodegenerative changes, enhance staging and stratification frameworks, and inform clinical trial enrichment targeting early tau‐related neurodegeneration.

## 2 METHODS

### 2.1 Participants

Participants were selected from the Pennsylvania Alzheimer's Disease Research Center Aging Brain Cohort (ABC) study. Inclusion criteria were the availability of a T1‐weighted MRI scan, at least one tau biomarker (tau‐PET or plasma p‐tau217), and a known Aβ status based on Aβ‐PET or plasma p‐tau217/Aβ_42_ ratio. Because tau biomarkers were collected at variable time points, analyses were anchored to the tau assessment. For participants with multiple tau–MRI pairings, we selected the MRI scan with the shortest absolute interval from the tau measurement. Both CU and cognitively impaired (CI) individuals were included. Cognitive status was based on multidisciplinary consensus diagnosis.^27^ All participants provided written informed consent in accordance with the Declaration of Helsinki under protocols approved by the University of Pennsylvania Institutional Review Board.

### 2.2 Image acquisition and processing

#### 2.2.1 Image acquisition

MRI scans were acquired on a Siemens Prisma 3T MRI scanner at the University of Pennsylvania. T1‐weighted structural images were obtained with a magnetization‐prepared gradient‐echo (MPRAGE) sequence using the following parameters: repetition time = 2400 ms, echo time = 2.24 ms, inversion time = 1060 ms, flip angle = 8°, 208 axial slices, slice thickness = 0.8 mm, field of view = 240 × 256 mm, and in‐plane resolution = 0.8 mm × 0.8 mm.

Aβ‐PET imaging was acquired using four 5‐min frames from 90 to 110 min after injection of 8.1 mCi ± 10% ^18^F‐florbetaben or 50 to 70 min after injection of 10 mCi ± 10% ^18^F‐florbetapir. Tau‐PET imaging was acquired using six 5‐min frames 75 to 105 min after the injection of 10 mCi ± 10% ^18^F‐flortaucipir. PET images were acquired on a Phillips Ingenuity TF PET/CT scanner.

#### 2.2.2 T1‐MRI processing

MTL subregion segmentation was performed using the ASHS‐T1 pipeline (https://sites.google.com/view/ashs‐dox/), with a newly defined atlas extending further into the anterior MTL.^25^ This extended atlas provides expanded coverage of anterior medial temporal lobe regions, including detailed delineation of ERC and BA35, and inclusion of the amygdala, which were not fully captured in earlier ASHS implementations. A visual comparison of the prior and extended atlas is provided in Figure S1. Segmented regions included anterior and posterior hippocampus (AH, PH), amygdala, ERC, BA35, BA36, and parahippocampal cortex (PHC). ASHS‐T1 is optimized for the MTL and minimizes confounds such as dura mater inclusion that affect whole‐brain parcellation tools, using a multi‐atlas label fusion approach in which each scan is non‐linearly registered to multiple expert‐labeled atlases, and the consensus segmentation is refined with boundary correction to improve subregional accuracy.^24^ In addition, the atlas includes a label for the choroid plexus, enabling quantification of off‐target tau‐PET signal in adjacent medial temporal regions.

For volumetric analyses, region‐of‐interest (ROI) volumes were extracted for AH, PH, and amygdala using ASHS‐T1 segmentations. For cortical regions (ERC, BA35, BA36, PHC), median thickness was computed using the cortical reconstruction for ASHS (CRASHS) surface‐based pipeline as previously described.^22^ Briefly, ASHS‐T1 provides anatomically precise volumetric segmentations of MTL subregions that serve as the basis for both ROI‐level summary measures and surface‐based cortical modeling. CRASHS builds directly on these ASHS‐T1 segmentations by generating participant‐specific gray/white, pial, and mid‐surfaces of the MTL cortex as triangular meshes, which are used to estimate cortical thickness. To enable group‐level morphometric analysis, individual MTL surfaces were aligned to a population template using diffeomorphic surface registration. This framework enables the derivation of both median cortical thickness estimates for each ROI and spatially resolved, vertex‐wise thickness maps with consistent anatomical correspondence across participants, allowing vertex‐wise statistical analyses of localized disease effects.

All subregion segmentations underwent visual quality control (QC). Each MTL subregion was assessed separately, and if an error was identified, only that subregion was excluded from all analyses while other regions were retained. Across all participants and hemispheres, failures were most frequent in the PH (12.6%) and ERC (10.0%), followed by PHC (8.9%), BA36 (4.2%), BA35 (4.6%), and AH (2.8%) due to oversegmentation or undersegmentation. No failures were observed in the amygdala. Across the seven ROIs examined, only two participants had failed segmentations in more than five regions. When examining participant groups, 12.2% of all QC failures occurred in CI participants and 6.9% in CU participants. Overall, segmentation quality was high.

Intracranial volume (ICV) was estimated from each participant's structural MRI using in‐house segmentation software built on the ASHS framework.^24^ Additionally, T1‐weighted scans were bias‐corrected and skull‐stripped using Advanced Normalization Tools (ANTs), followed by cortical and subcortical parcellation with multi‐atlas Joint Label Fusion and the BrainColor atlas, which defines 102 cortical ROIs.^28^, ^29^ These parcellations were used to derive cerebellar and cortical reference region segmentations for subsequent PET normalization.

#### 2.2.3 PET processing

PET acquisition and processing followed an in‐house pipeline modeled after Alzheimer’s Disease Neuroimaging Initiative (ADNI) procedures, consistent with our prior work.^30^ For tau‐PET, six 5‐min frames acquired 75 to 105 min after injection of ^18^F‐flortaucipir were realigned to the first frame, averaged, resampled into 1.5‐mm^3^ AC–PC–aligned space, and smoothed to a uniform 6‐mm full‐width at half maximum (FWHM) resolution.

All PET volumes were rigidly registered to each participant's T1‐weighted MRI using ANTs,^31^ and all registrations were visually inspected for QC using standardized slice and grid overlays. Standardized uptake value ratio (SUVR) maps were generated using an inferior cerebellar reference region for tau‐PET. For tau‐PET analyses, mean SUVR was extracted from the combined ERC and BA35 from the ASHS‐T1 atlas, as a summary measure of MTL tau load. This region was selected based on extensive neuropathological and imaging evidence demonstrating that tau accumulation originated in BA35 and the ERC before spreading posteriorly along the MTL axis. Accordingly, ERC/BA35 SUVR was used as an index of early tau burden to test whether pathology in these earliest‐affected regions was associated with spatial patterns of neurodegeneration across the MTL. For sensitivity analyses, ROI‐wise and vertex‐wise SUVR values were also extracted to evaluate localized associations. However, interpretation of these analyses is limited by the spatial resolution of PET imaging relative to the size of MTL subregions, especially thin cortical structures such as ERC and BA35, as well as the off‐target binding from the choroid plexus, which was explicitly segmented using the ASHS‐T1 atlas.

### 2.3 Plasma biomarkers

Plasma p‐tau217 and Aβ_42_ levels were measured using the Fujirebio Lumipulse platform. All analyses were conducted on a Fujirebio Lumipulse G1200 analyzer at the University of Pennsylvania.

### 2.4 Amyloid status determination

Aβ status was determined using PET or plasma biomarkers. For participants with Aβ‐PET, Aβ status was based on visual read by an experienced nuclear medicine physician/neuroradiologist (I.M.N). For participants without Aβ‐PET, Aβ status was defined by the p‐tau217/Aβ_42_ ratio, with a cutoff of >0.0055 indicating positivity. This threshold corresponds to 95% sensitivity and ∼90% classification accuracy relative to Aβ‐PET.^32^

### 2.5 Statistical analysis

All statistical analyses were conducted in R version 4.4.2 (www.r‐project.org), except for the vertex‐wise MTL analyses, which were performed in Python using the CM‐Rep package (github.com/pyushkevich/cmrep). Scripts used for analysis are available upon request.

Participants were stratified into three analysis groups: (1) CU Aβ−, (2) CU Aβ+, and (3) All Aβ+ (CU Aβ+ and CI Aβ+ combined). This stratification was chosen to (i) isolate early tau‐related structural changes in CU Aβ+ individuals, a population of particular interest for preclinical disease characterization and clinical trial enrichment, and (ii) examine associations across the full spectrum of Aβ+ individuals, increasing the dynamic range of biomarker and structural measures and improving sensitivity to detect structure–tau relationships. Exploratory analyses restricted to the CI Aβ+ subgroup were also performed as they may provide complementary clinical stage‐specific context and are presented in the Supplementary Materials for completeness. The CU Aβ− group was included as a reference to contextualize findings in the Aβ+ groups, serving as a comparison group for evaluating sensitivity of tau‐related associations.

Two CU Aβ− participants were excluded from plasma p‐tau217 analyses. One was excluded due to a spurious plasma p‐tau217 value (>4 pg/µL). Another was identified as an outlier based on high leverage in regression diagnostics. This individual had previously been noted as a possible PART case due to focal left anterior hippocampal atrophy and elevated MTL tau‐PET signal. Because this datapoint exerted disproportionate influence, this participant was excluded.

#### 2.5.1 Spatial distribution of tau‐PET SUVR

To characterize the spatial pattern of tau deposition, tau‐PET SUVR maps were sampled along the mid‐surface of the MTL using the MeshImageSample tool from the CM‐Rep package. Mean SUVR maps were generated for each group to qualitatively assess the anterior–posterior gradient of tau accumulation. These maps were used to illustrate group‐wise differences in the distribution of tau signal across anterior (AH, ERC, BA35) and posterior (PH, BA36, PHC) MTL regions.

#### 2.5.2 Associations between vertex‐wise thickness and tau measures

We next performed vertex‐wise analyses using the meshglm tool from the CM‐Rep package to assess localized associations between tau biomarkers and cortical thickness across the MTL surface (not including amygdala). For each vertex, a general linear model (GLM) was fit separately for tau‐PET and plasma p‐tau217. All models included age and sex as covariates:

$$\begin{array}{ccc} & & \text{Tau}-\text{PET}\;\text{models}:\text{Thickness}∼\text{Ipsilateral}\;\text{ERCBA}35\;\text{SUVR}+\text{Age}+\text{Sex}\\ & & \text{Plasma}\;p-\text{tau}217\text{models}:\text{Thickness}∼p-\text{tau}217+\text{Age}+\text{Sex} \end{array}$$

Statistical inference at each vertex was carried out using non‐parametric permutation testing (10,000 permutations) with the threshold‐free cluster enhancement (TFCE) method^33^ (parameters: *E* = 0.5, *H* = 2, Δ*h* = 0.01) to identify clusters without requiring an arbitrary threshold. Family‐wise error rate (FWER)‐corrected *p* values were computed to control for multiple comparisons across the surface.

#### 2.5.3 Associations between regional volume/thickness and tau biomarkers

We assessed associations between regional morphometry and tau measures using partial Spearman's rank correlations to accommodate potential non‐normality in imaging and plasma variables, controlling for age, sex, and ICV (via the ppcor package in R). Partial Spearman correlation coefficients (ρ) were estimated, and 95% confidence intervals were derived using non‐parametric bootstrap resampling (1000 iterations). Regional volumes (AH, PH, amygdala) and cortical thickness ROIs (ERC, BA35, BA36, PHC) were tested against two tau biomarkers: (1) mean ERC/BA35 tau‐PET SUVR and (2) plasma p‐tau217 concentration. To account for multiple comparisons across ROIs, we applied false discovery rate (FDR) correction using the Benjamini–Hochberg procedure, performed separately within each biomarker × group analysis. Because the tau‐PET analyses were limited by sample size, FDR correction may be overly conservative and obscure biologically meaningful effects. Therefore, results are reported at an uncorrected threshold in the main text and figures, with FDR‐corrected results provided in the supplementary files.

To determine whether specific ROIs showed stronger associations with tau relative to others, we performed Hittner's *Z* tests for comparing overlapping dependent correlations (via the cocor package in R). This test accounts for the shared variance between ROIs when comparing their respective correlations with a common biomarker. Results are reported as Z statistics with corresponding two‐tailed *p* values.

#### 2.5.4 Sensitivity analyses

For tau‐PET to structure analyses as in Sections 2.5.2 and 2.5.3, complementary vertex‐to‐vertex and matched ROI‐to‐ROI analyses were conducted as sensitivity analyses to assess the robustness of findings to the choice of tau measure (see Supplementary Materials). These analyses examine spatially localized associations between tau‐PET signal and structural measures but are limited by the spatial resolution of PET relative to the size of MTL subregions and by partial volume effects, particularly in thin cortical regions, as well as the off‐target binding from the choroid plexus. For this reason, they were not used as primary analyses. In addition to the covariates included in the primary models, choroid plexus SUVR was included as a covariate to account for potential off‐target binding. Model equation:

$$\begin{array}{ccc} \text{Vertex}/\text{ROI}\;\text{thickness} & ∼ & \text{Vertex}/\text{ROI}\text{SUVR}\;+\text{Age}+\text{Sex}\\ & & +\;\text{Choroid}\;\text{plexus}\text{SUVR} \end{array}$$

## 3 RESULTS

Summary characteristics of the groups are reported in Table 1. We analyzed 448 unique participants (246 CU Aβ−, 79 CU Aβ+, 123 CI Aβ+), of whom 199 had tau‐PET and 377 had plasma p‐tau217 available. Participants were split into three groups for analyses: (1) CU Aβ−, (2) CU Aβ+, and (3) All Aβ+ (39% CU Aβ+, 61% CI Aβ+). Groups were similar in demographic characteristics, but, as expected, higher MTL tau‐PET SUVR and plasma p‐tau217 levels were observed in the Aβ+ groups. Table S1 shows group characteristics broken down by CU Aβ−, CU Aβ+, CI Aβ+, and All Aβ+.

**TABLE 1:** Participant characteristics by analysis group.

	CU Aβ− (*n* = 246)	CU Aβ+ (*n* = 79)	All Aβ+ (*n* = 202)
Age, years	73.0 ± 5.9	75.6 ± 6.7	74.9 ± 7.2
Sex, female (%)	66.2	69.6	57.4
Education, years	16.3 ± 2.6	16.4 ± 2.4	16.2 ± 2.7
Race (White/Black/2+)	145/92/4	53/21/2	151/39/3
CU/MCI/dementia	246/0/0	79/0/0	79/64/59
ERC/BA35 tau‐PET SUVR	1.07 ± 0.10 (*n* = 129)	1.21 ± 0.21 (*n* = 32)	1.38 ± 0.27 (*n* = 70)
Time between tau‐PET and MRI (days)	164.6 ± 152.5	236.1 ± 182.7	158.0 ± 176.8
Plasma p‐tau217, pg/µL	0.11 ± 0.11 (*n* = 198)	0.27 ± 0.14 (*n* = 67)	0.52 ± 0.43 (*n* = 179)
Time between plasma p‐tau217 and MRI (days)	63.1 ± 84.2	60.8 ± 63.6	82.3 ± 83.9
CDR sum of boxes	0.04 ± 0.16 (*n* = 233)	0.12 ± 0.34 (*n* = 68)	2.57 ± 3.24 (*n* = 187)
MoCA	26.5 ± 2.4 (*n* = 234)	26.4 ± 2.7 (*n* = 68)	21.5 ± 6.1 (*n* = 188)

*Note*: Values are presented as mean ± standard deviation (SD) for continuous measures and percent for categorical variables. Sex is shown as percentage female. Race shown as counts for White, Black or African American, and more than two races. For biomarker measures, the number of participants with available data is indicated in parentheses.

Abbreviations: CU Aβ−, cognitively unimpaired amyloid beta‐negative; CU Aβ+, cognitively unimpaired amyloid beta‐positive; All Aβ+, all amyloid beta‐positive participants (CU Aβ+ and CI Aβ+ combined); ERC, entorhinal cortex; BA35, Brodmann area 35; SUVR, standardized uptake value ratio.

### 3.1 Pattern of tau‐PET SUVR in the MTL

**FIGURE 1:**
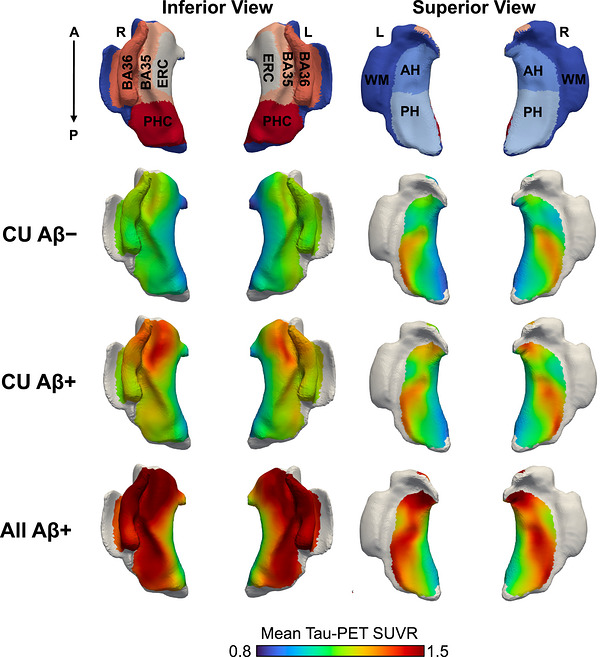
Spatial gradient of tau‐PET SUVR across MTL surface. Mean tau‐PET SUVR maps projected onto the MTL surface illustrate the distribution of tracer uptake across CU Aβ−, CU Aβ+, and All Aβ+ groups. A clear anterior–posterior gradient was observed, with greatest signal in the anterior hippocampus, ERC, and BA35 and relatively lower uptake in posterior hippocampus and PHC. This gradient was not present in CU Aβ−, emerged in CU Aβ+, and was most pronounced in the All Aβ+ group. These qualitative patterns underscore the anterior MTL as a key locus of early tau deposition, complementing ROI‐ and vertex‐level analyses. Note: The elevated signal along the superior hippocampal surface likely reflects off‐target choroid plexus binding. CU Aβ− = cognitively unimpaired amyloid beta‐negative; CU Aβ+ = cognitively unimpaired amyloid beta‐positive; All Aβ+ = all amyloid beta‐positive participants (CU Aβ+ and CI Aβ+ combined); ERC = entorhinal cortex; BA35/36 = Brodmann area 35/36; PHC = parahippocampal cortex; AH = anterior hippocampus; PH = posterior hippocampus; WM = white matter; SUVR = standardized uptake value ratio.

Surface maps of mean tau‐PET SUVR revealed a consistent anterior–posterior gradient of tracer uptake across groups, with highest signal in AH, ERC, and BA35 and relatively lower uptake in PH and PHC (Figure 1). Visually, CU Aβ+ participants demonstrated a discernible anterior predominance, with localized increases in ERC and BA35 relative to posterior regions. The gradient was most pronounced in the All Aβ+ group, where elevated uptake extended broadly across AH and ERC and into BA35, while posterior regions showed comparatively modest involvement. And as expected, in the CU Aβ− individuals, average uptake was minimal across the MTL surface with no clear gradient. Together, these group‐level maps highlight the emergence of anterior‐predominant tau deposition in CU Aβ+, which becomes more widespread and robust in the full Aβ+ group, consistent with early Braak stage progression. These qualitative patterns underscore the anterior MTL, especially the anterior parts of MTL cortical regions recently added to the ASHS‐T1 pipeline, as a key locus of early tau deposition, complementing the ROI‐ and vertex‐level analyses. The restricted CI Aβ+ group showed increased SUVR throughout the MTL cortex and PH (Figure S2A). CU Aβ− and CU Aβ+ referenced z‐scored maps are shown in Figure S2B,C and further illustrate the anterior predominance in early disease followed by more diffuse MTL involvement in clinical stages.

### 3.2 Associations between vertex‐wise thickness and tau measures

**FIGURE 2:**
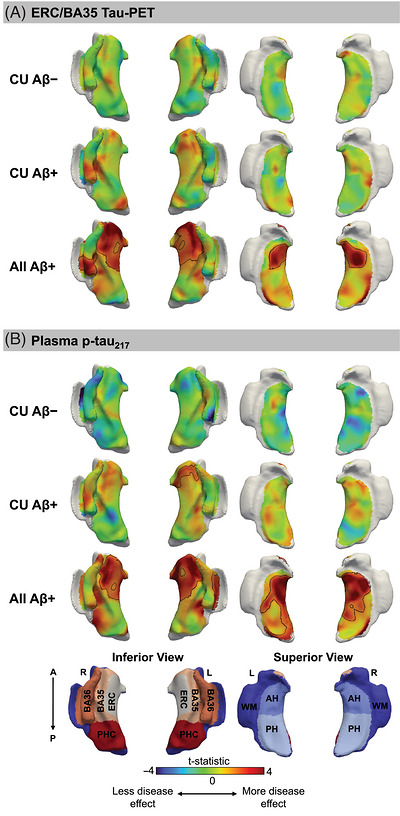
Vertex‐wise correlations of thickness with tau biomarkers across the MTL. Vertex‐wise maps display associations between cortical thickness and tau biomarkers (A: tau‐PET ROI; B: plasma p‐tau217) within CU Aβ−, CU Aβ+, and All Aβ+ groups. General linear models were fit at each vertex with age and sex as covariates. Significance was determined using permutation testing (10,000 permutations) with threshold‐free cluster enhancement (TFCE) and family‐wise error rate (FWER) correction. Black outlines represent significant clusters (*p* < 0.05). Both tau biomarkers demonstrated convergent clusters of negative correlations in anterior hippocampus, ERC, and BA35, with plasma p‐tau217 showing additional effects in posterior hippocampal regions. CU Aβ− = cognitively unimpaired amyloid beta‐negative; CU Aβ+ = cognitively unimpaired amyloid beta‐positive; All Aβ+ = all amyloid beta‐positive participants (CU Aβ+ and CI Aβ+ combined); ERC = entorhinal cortex; BA35/36 = Brodmann area 35/36; PHC = parahippocampal cortex; AH = anterior hippocampus; PH = posterior hippocampus; WM = white matter.

Vertex‐wise analyses revealed distinct spatial patterns of association between regional MTL morphometry and tau biomarkers across groups (Figure 2). In the CU Aβ+ group, higher tau‐PET SUVR was linked to localized patterns of thinner cortex and inward surface deformations in anterior regions, with subtle clusters in the ERC and BA35, that did not reach significance potentially due to the relatively small sample size. By contrast, plasma p‐tau217 levels were significantly associated with anterior cortical thinning in ERC and BA35, consistent with these regions being early sites of tau‐related structural vulnerability.^2^ In the broader All Aβ+ group, widespread negative associations were observed with both biomarkers, extending across AH and BA35, with additional significant clusters in ERC and PH. The spatial extent of these effects differed between modalities: tau‐PET associations were focal, with robust clusters concentrated along the anterior and medial hippocampal surface, and plasma p‐tau217 showed a broader distribution of effects extending into PH as well. In contrast, the CU Aβ− group showed no significant clusters for either biomarker. The restricted CI Aβ+ group also showed no significant correlations, potentially due to limited statistical power and a smaller dynamic range in the different biomarkers (Figure S3).

### 3.3 Associations between regional volume/thickness and tau measures

**FIGURE 3:**
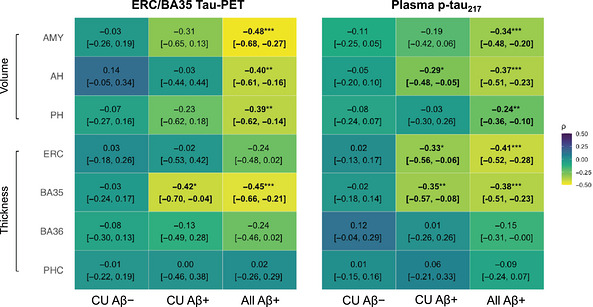
ROI‐wise correlations with tau biomarkers. Heatmaps of partial Spearman's correlations (ρ), controlling for age, sex, and ICV, between regional MTL measures (amygdala [AMY] anterior hippocampus [AH], posterior hippocampus [PH], entorhinal cortex [ERC], Brodmann Area 35 [BA35], BA36, parahippocampal cortex [PHC]) and tau biomarkers. Correlation coefficients are shown with 95% confidence intervals estimated using bootstrap resampling (*R* = 1000). Results are shown for ERC/BA35 tau‐PET SUVR (left) and plasma p‐tau217 (right), stratified by CU Aβ−, CU Aβ+, and All Aβ+ groups. Values inside tiles show correlation coefficients (ρ) (bolded when significant at *p < 0.05, **p < 0.01, ***p < 0.001). A consistent pattern of stronger associations in BA35, ERC, and hippocampal subregions is observed, with plasma p‐tau217 showing robust effects across groups. False discovery rate–corrected results are provided in Figure S5.

ROI‐wise partial correlations revealed consistent negative associations between anterior MTL integrity and both tau‐PET SUVR and plasma p‐tau217 levels when controlling for age, sex, and ICV, with effects most prominent in the amygdala, hippocampus, ERC, and BA35 (Figure 3; see scatterplots in Figure S4). Among CU Aβ+ participants, associations were confined to anterior regions, with higher ERC/BA35 tau‐PET SUVR correlating with thinner BA35 (*ρ* = −0.42), and plasma p‐tau217 showing similar effects across AH, ERC, and BA35 (ρ ≈ −0.29 to −0.35). In the broader All Aβ+ group, these associations not only persisted but were stronger and more widespread, with significant correlations observed across all anterior ROIs for both modalities (e.g., tau‐PET: amygdala *ρ* = −0.48, AH ρ = −0.40, BA35 ρ = −0.45; plasma p‐tau217: amygdala ρ = −0.34, AH ρ = −0.37, ERC ρ = −0.41, BA35 ρ = −0.38; all *p* < 0.001). By contrast, the CU Aβ− group showed no robust correlations with either tau biomarker. Collectively, these findings highlight convergent biomarker relationships, particularly for BA35. Notably, the more restricted pattern of significant associations observed for tau‐PET in CU Aβ+ individuals after covarying for age, sex, and ICV likely reflects the stronger age dependence of PET signal relative to plasma p‐tau217, as well as modest differences in sample size between modalities.

After applying FDR correction, all significant associations between plasma p‐tau217 and regional MTL measures remained unchanged, underscoring the robustness of plasma findings (Figure S5). In contrast, for tau‐PET, the previously observed associations in the CU Aβ+ group with BA35 thickness was no longer significant following FDR adjustment, while effects in the All Aβ+ group remained robust. No significant associations were detected within the restricted CI Aβ+ (Figure S6).

We then formally compared the strength of the associations across different regions using the Hittner's *Z* test, conducted separately within volumetric ROIs (AH, PH, amygdala) and thickness ROIs (ERC, BA35, BA36, PHC). Comparisons were limited to ROI pairs in which at least one region demonstrated a significant association with tau‐PET SUVR or plasma p‐tau217, ensuring that tests were restricted to biologically meaningful effects (Table S2). In CU Aβ+, BA35 showed stronger associations than PHC (*Z* = 2.20, *p* = 0.03) for tau‐PET and stronger associations than PHC (*Z* = 2.80, *p* = 0.005) and BA36 (*Z* = 2.31, *p* = 0.02) for plasma p‐tau217. ERC showed stronger associations than PHC (*Z* = 2.13, *p* = 0.03) for plasma p‐tau217. In the broader All Aβ+ group, BA35 again emerged as a particularly sensitive locus, with tau‐PET correlations stronger than ERC (*Z* = 2.40, *p* = 0.01), PHC (*Z* = 4.36, *p* < 0.001) and BA36 (*Z* = 2.76, *p* = 0.005), and plasma p‐tau217 correlations stronger than PHC (*Z* = 4.00, *p* < 0.001) and BA36 (*Z* = 2.92, *p* = 0.003). ERC showed stronger associations than PHC (*Z* = 3.44, *p* < 0.001) and BA36 (*Z* = 2.76, *p* = 0.006) for plasma p‐tau217. No other ROI pairs showed significant differences across groups.

Taken together, these tests complement the correlation results by confirming that BA35 consistently demonstrates stronger correlations with tau biomarkers relative to neighboring ERC and PHC, reinforcing its role as an early vulnerable region.

### 3.4 Sensitivity analyses

Repeated vertex–vertex and ROI–ROI analyses with tau‐PET SUVR and thickness analyses yielded consistent spatial patterns of association. Vertex‐to‐vertex correlations showed similar patterns with a more anterior predominance within the All Aβ+ group, although with less extensive clusters (Figure S5). Matched tau‐PET ROI SUVR to matched structural ROI metric correlations are shown in Figure S6, which produced largely similar results within the CU Aβ+ and All Aβ+ groups.

## 4 DISCUSSION

In this study, we applied a granular segmentation approach to the MTL to examine early, regionally specific tau‐related neurodegeneration in AD. The present work extends prior studies by combining a histologically informed, anteriorly extended MTL segmentation with multimodal tau biomarkers to detect early, spatially restricted neurodegeneration in anterior MTL regions and reveal convergent patterns across tau‐PET and plasma p‐tau217. For both PET and plasma, associations with atrophy followed a gradient toward the anterior MTL, with particularly strong associations in BA35 and ERC, confirming that these regions show the earliest and most specific structural signatures of tau‐related neurodegeneration in vivo. Importantly, this gradient was most pronounced in CU Aβ+ individuals.

The anatomically detailed segmentation approach used here delineates fine subdivisions of key cortical territories, including BA35 and the ERC, and extends coverage to more anterior regions than prior atlas implementations. Few existing segmentation tools explicitly define BA35 or adequately sample these anterior territories, resulting in underrepresentation of the zones most susceptible to early pathology. By incorporating anterior extent and anatomically defined subregions, our approach enables anatomically precise mapping of subregional vulnerability that mirrors the progression of NFT accumulation observed in neuropathological staging and allows for the detection of focal neurodegeneration in preclinical disease. Notably, prior ASHS implementations^18^ or other parcellation approaches^6^, ^14^, ^15^ did not fully capture the anterior MTL territories, which we show exhibit detectable preclinical neurodegeneration.

Our tau‐PET spatial gradient analyses revealed that in CU Aβ+ individuals, tracer uptake was highest in BA35 and ERC, the regions showing strongest structural associations. These territories correspond to the earliest cortical sites of tau deposition described in *post mortem* studies,^1^, ^3^, ^4^ including recent quantitative analyses of regional tau density,^10^ as well as confirmed in vivo by recent tau‐PET work demonstrating that tau accumulation originates in transentorhinal and entorhinal cortices before spreading posteriorly along the MTL.^34^ The anterior‐to‐posterior gradient of tau burden observed across MTL subregions recapitulates this established pattern, demonstrating that when combined with anatomically precise, subregion‐level segmentation, tau‐PET can recover the fine spatial organization of early tau pathology in vivo. A band of elevated signal extending laterally and posteriorly along the superior MTL surface likely reflects off‐target tracer retention in the choroid plexus,^35^ a known characteristic of ^18^F‐flortaucipir. This off‐target tracer retention can be visualized and distinguished with our surface‐based framework. Together, these results show that anatomically faithful segmentation enhances the interpretability of regional tau‐PET data and enables the spatial gradients described in neuropathological staging to be resolved in preclinical disease in vivo.

Beyond tau distribution, our findings reveal tight coupling between tau burden and local neurodegeneration within the MTL. In CU Aβ+ individuals, tau‐related structural changes were largely confined to BA35 and anterior ERC. This topographic specificity converges with *post mortem* work showing that NFTs accumulate in the transentorhinal region before involving the hippocampus proper. Importantly, by restricting the analyses to CU Aβ+, we were able to isolate these earliest patterns, which became less focal when examining All Aβ+ individuals, where the neurodegenerative effects of tau pathology were more widely distributed across the MTL. These findings highlight the value of anteriorly extended segmentation for detecting subtle, stage‐specific changes that may otherwise be obscured in heterogeneous or more advanced disease cohorts. While we also examined associations within the CI Aβ+ subgroup, interpretation of these findings is difficult due to smaller sample size and increased likelihood of co‐occurring pathologies in clinically impaired individuals, which may confound associations between AD pathology and brain structure.^36^

Prior studies examining tau‐related structural changes in CU individuals reported relatively modest effect sizes and mixed findings. Importantly, several studies reporting associations in CU participants examined correlations across both Aβ− and Aβ+ individuals,^6^, ^14^, ^15^, ^18^ which may partly reflect a broader range of tau burden and disease stage. Consistent with this, large multi‐cohort analyses focusing on CU Aβ+ individuals have similarly reported limited cross‐sectional effects; for example, in a cohort of over 2000 preclinical AD participants, CU Aβ+ individuals without elevated tau (A+T−) showed no detectable cortical thinning cross‐sectionally, although longitudinal analyses revealed progressive ERC and BA35 atrophy as tau burden increased.^22^ In contrast, cross‐sectional atrophy within these same anterior MTL regions has been observed using higher‐resolution imaging, such as T2‐weighted morphometry in ADNI, which revealed early BA35 thinning in all Aβ+ individuals,^36^ and dedicated ERC analyses showing associations between local tau burden and entorhinal cortical thinning in preclinical AD.^6^, ^18^ The presence of cross‐sectional associations in our study, particularly within BA35 and ERC, suggests that a more granular segmentation framework is sensitive to early, spatially restricted neurodegeneration also using standard resolution imaging. By combining anatomically precise anterior MTL segmentation with surface‐based modeling that accounts for inter‐individual variability in MTL folding patterns (e.g., collateral sulcus morphology), our approach enables spatially resolved interrogation of focal atrophy beyond what is achievable with ROI‐level volumetric measures.

The biological relevance of this subregional specificity is supported by converging evidence from functional and histopathological studies. Functional imaging work has shown that the earliest alterations in preclinical AD involve disrupted connectivity within the anterior–temporal system, particularly between the entorhinal and perirhinal cortices (includes BA35) and their cortical partners, preceding detectable structural atrophy.^37^, ^38^ Complementary *post mortem* three‐dimensional mapping of NFT burden in the MTL has revealed transitions in tau density across neighboring regions, especially at the ERC–BA35–hippocampal boundaries.^39^, ^40^ These studies underscore the need for anatomically precise frameworks capable of capturing early, region‐specific vulnerability. Our results extend this evidence by demonstrating that these same subregions – BA35 and ERC – show focal tau‐related neurodegeneration in vivo, reinforcing the biological and translational value of granular MTL segmentation in studying the earliest stages of AD.

Our granular characterization of the MTL is relevant in light of the recently revised National Institute on Aging–Alzheimer's Association criteria for AD, which explicitly emphasizes medial temporal tau burden as a key region defining the biological stage “B” of AD.^41^ The criteria recognize that tau distribution within the MTL reflects the earliest stages of symptomatic risk and provides prognostic value beyond global tau burden. Large cohort analyses have confirmed that staging based on MTL tau stratifies individuals’ risk for decline,^19^, ^42^ and updated appropriate use criteria for PET also underscore the importance of regional tau measures for clinical decision‐making.^43^ Beyond enhancing detection of early tau‐PET changes, our approach offers a practical and scalable tool that complements Core 2 tau‐PET and Core 1 plasma measures, which reflect distinct aspects of tau pathology, directly situating MRI‐based anterior MTL metrics within the evolving diagnostic framework.

From a translational perspective, this framework holds particular promise for clinical trial design and monitoring. The ability to localize tau‐related atrophy to anterior MTL cortices in CU Aβ+ individuals suggests a potential role for these measures in enriching preclinical or prodromal trial cohorts, identifying those most likely to progress.^19^, ^44^ Moreover, the atlas's reproducible and anatomically grounded subregions provide a sensitive method for assessing disease‐modifying therapy effects in focal MTL territories impacted early in the AD neuropathologic timeline. Together, these applications position granular MTL segmentation as a bridge between mechanistic understanding of early AD and its implementation in biomarker‐guided prevention and intervention studies.

While we examined the tau‐related neurodegeneration in the present study, the anterior regions impacted in early AD are also vulnerable to TAR DNA binding protein 43 (TDP‐43) pathology.^45^, ^46^, ^47^, ^48^, ^49^ Thus, in the absence of specific markers of TDP‐43 pathology, we cannot exclude the potential impact of co‐pathology on these associations. However, the use of granular, anatomically precise segmentation of anterior MTL subregions (e.g., ERC and BA35) enables these regions to be examined separately, providing a framework to better characterize overlapping patterns of tau‐ and TDP‐43–related neurodegeneration. This approach may facilitate identification of mixed‐pathology signatures in vivo^50^ and inform studies of related neurodegenerative syndromes where anatomical precision is critical.

Our study has several potential limitations to acknowledge. First, our analyses were cross‐sectional, limiting inferences about longitudinal disease processes. Nonetheless, our analyses examined regional neurodegeneration as a function of continuous tau burden rather than discrete group contrasts, and this design partially captures progression along the AD continuum. Second, although tau‐PET and plasma p‐tau217 are highly correlated even after controlling for amyloid pathology, they reflect subtly different stages of tau pathology, which, along with differences in sample sizes, may account for the differences between the two measures. Additionally, ^18^F‐flortaucipir is subject to off‐target binding in the choroid plexus, which can influence signals in adjacent MTL regions. However, sensitivity analyses controlling for choroid plexus signal yielded similar effects in the anterior MTL. Finally, larger and more demographically diverse samples will be needed to confirm generalizability and to further validate these methods across diverse populations and disease contexts.

In summary, this study demonstrates that granular segmentation of the MTL enhances measurement of early tau‐related neurodegeneration and captures the spatial progression of tau pathology observed in *post mortem* studies. By combining anatomically precise structural measures with molecular biomarkers, this framework offers a biologically grounded and clinically translatable approach for probing preclinical AD. The ability to resolve subregional patterns within the MTL provides a powerful foundation for studying disease heterogeneity, refining diagnostic staging, and extending these insights to related neurodegenerative diseases – ultimately advancing efforts to detect, monitor, and treat AD before the onset of clinical symptoms.

## CONFLICT OF INTEREST STATEMENT

Nidhi S. Mundada, Niyousha Sadeghpour, Emily McGrew, Hannah L. Tucker, llya M. Nasrallah, Paul A. Yushkevich, Christopher A. Brown, and Laura E.M. Wisse declare no competing interests. Sandhitsu R. Das has received consulting fees from Rancho Biosciences and NIA Therapeutics. David A. Wolk has served as a paid consultant for Eli Lilly and Beckman Coulter. He has also served on the Data and Safety Monitoring Board for GSK. He has received research support paid to his institution by Biogen. Author disclosures are available in the Supporting Information.

## CONSENT STATEMENT

We confirm that all human subjects provided informed consent. Human brain specimens were obtained in accordance with the State of Pennsylvania and University of Pennsylvania Institutional Review Board guidelines. Where possible, pre‐consent during life and, in all cases, next‐of‐kin consent at death was given.

## Supporting information



Supporting Information: alz71571‐sup‐0001‐SuppMat

Supporting Information: alz71571‐sup‐0002‐ICMJE
