# Supplementary material for: Vulnerability of anterior medial temporal lobe subregions to early tau‐related neurodegeneration in Alzheimer's disease: Converging evidence from tau‐PET and plasma p‐tau217
Source: Alzheimers Dement. 2026 Jun 17;22(6):e71571. doi: 10.1002/alz.71571 (PMC13275324; doi:10.1002/alz.71571)
Supplement: Supplementary file 1 — Supporting Information: alz71571‐sup‐0001‐SuppMat [file ALZ-22-e71571-s002.docx]

**
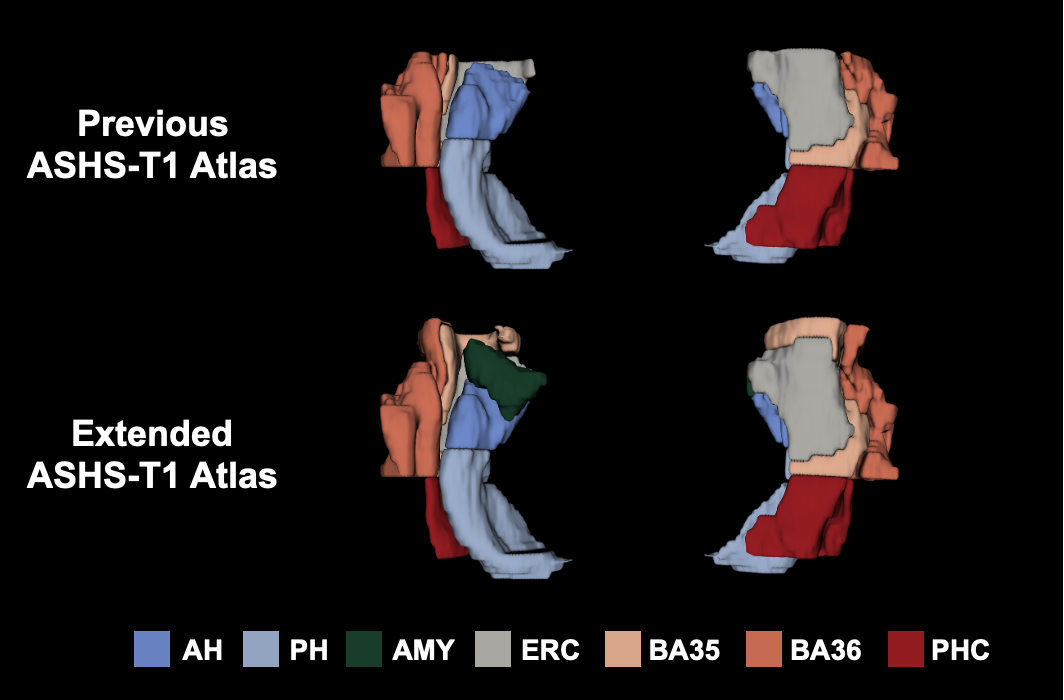
**

**Supplementary Figure 1. Comparison of MTL regions included in prior ASHS segmentation versus the extended ASHS atlas**

*The original ASHS-T1 atlas (top) compared to the extended ASHS-T1 atlas (bottom) which provides expanded anterior MTL coverage (in the ERC, BA35, BA36) and includes additional subregions (amygdala).*

**
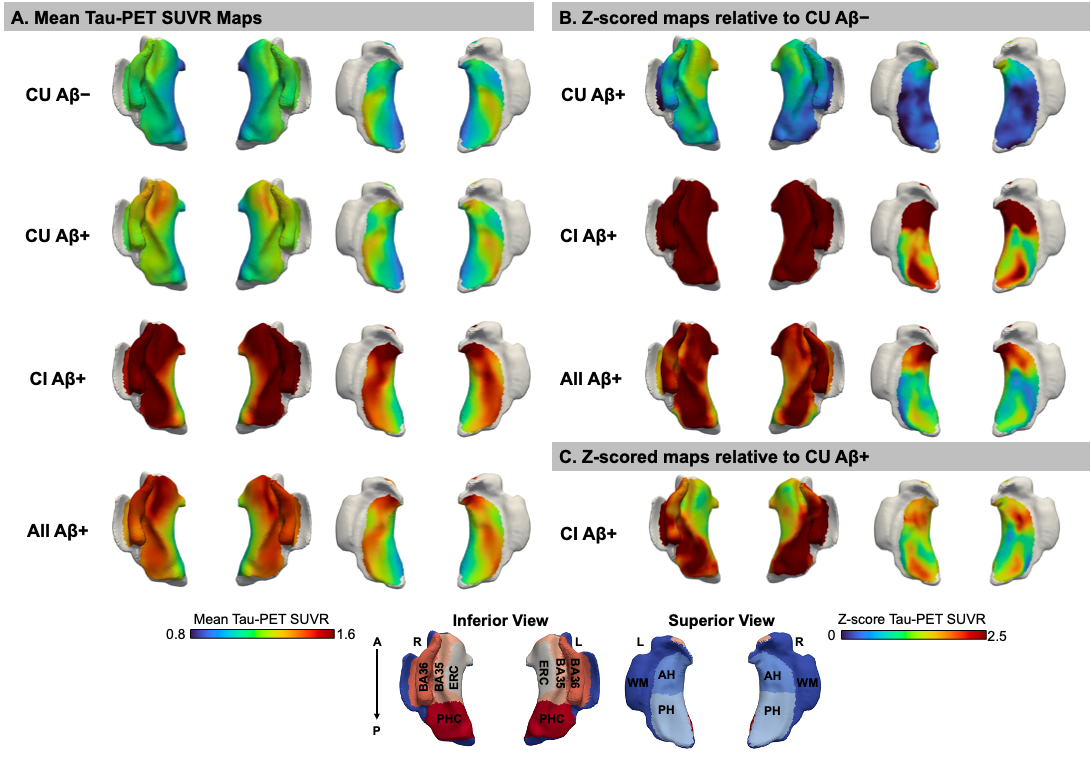
**

**Supplementary Figure 2. Spatial gradient of mean and z-scored tau-PET SUVR across the MTL surface**

*A. Mean tau-PET SUVR maps projected onto the MTL surface illustrate the distribution of tracer uptake across CU Aβ−, CU Aβ+, CI Aβ+, and All Aβ+ groups. B. Z-scored tau-PET SUVR maps projected onto the MTL surface illustrate the distribution of tracer uptake across CU Aβ+, CI Aβ+ and All Aβ+ groups relative to CU Aβ−. A clear anterior–posterior gradient was observed, with greatest signal in the anterior hippocampus, ERC, and BA35, and relatively lower uptake in posterior hippocampus and PHC. C. Z-scored tau-PET SUVR maps projected onto the MTL surface illustrate the distribution of tracer uptake across CI Aβ+ group relative to CU Aβ+. A relatively lower z-score in ERC and BA35 in CI Aβ+ when referenced to CU Aβ+, suggesting that tau-PET signal in these regions may reach a plateau early in the disease course, potentially prior to symptom onset. CU Aβ−= cognitively unimpaired amyloid-β-negative; CU Aβ+ = cognitively unimpaired amyloid-β-positive; CI Aβ+ = cognitively impaired amyloid-β-positive participants; All Aβ+ = all amyloid-β-positive participants (CU Aβ+ and CI Aβ+ combined); ERC = entorhinal cortex; BA35/36 = Brodmann area 35/36; PHC = parahippocampal cortex; AH = anterior hippocampus; PH = posterior hippocampus; WM = white matter; SUVR = standardized uptake value ratio.*

*
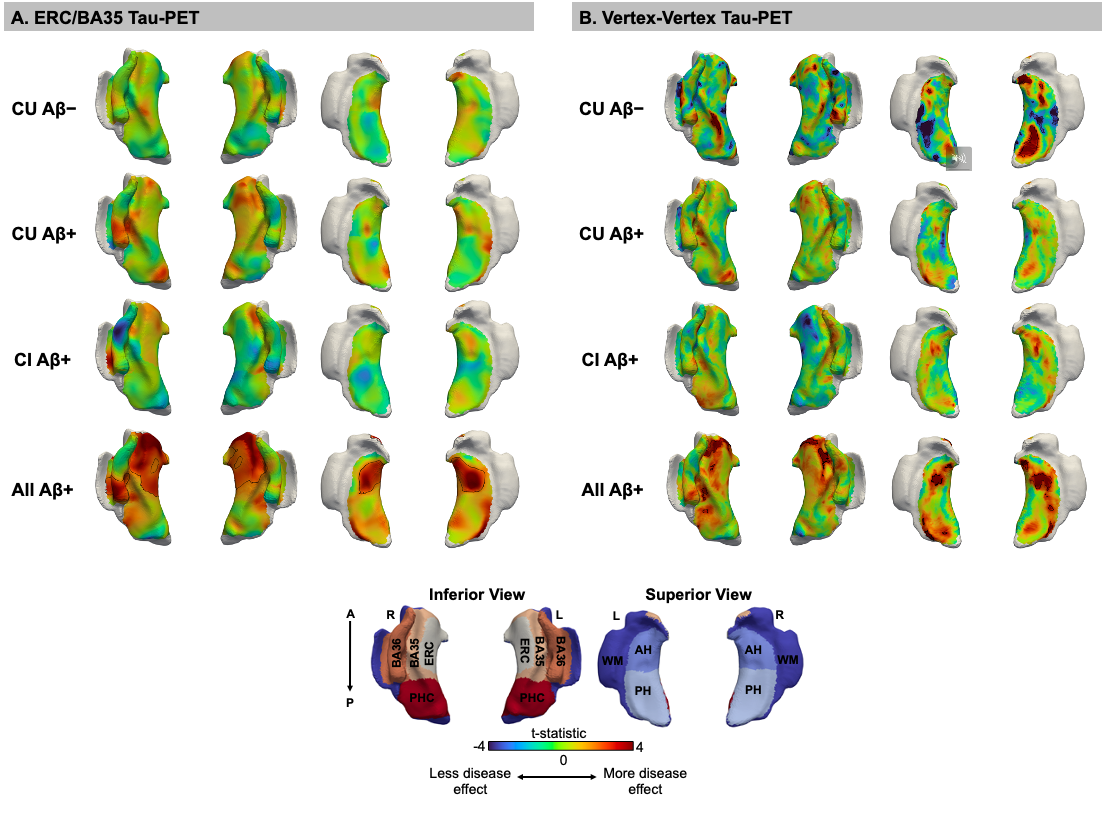
*

**Supplementary Figure 3. Vertex-wise correlations of thickness with Tau PET (ERC/BA35 SUVR versus vertex-based SUVR).**

*Vertex-wise maps display associations between cortical thickness and tau PET (A: ERC/BA35 Tau-PET ROI; B: vertex-wise Tau-PET SUVR) within CU Aβ−, CU Aβ+, CI Aβ+, and All Aβ+ groups. General linear models were fit at each vertex with age and sex as covariates. Significance was determined using permutation testing (10,000 permutations) with threshold-free cluster enhancement (TFCE) and family-wise error rate (FWER) correction. Black outlines represent significant clusters (p < 0.05). Age and sex were used as covariates for (A) and in addition to mean choroid-plexus tau-PET SUVR for (B). CU Aβ−= cognitively unimpaired amyloid-β-negative; CU Aβ+ = cognitively unimpaired amyloid-β-positive; CI Aβ+ = cognitively impaired amyloid-β-positive; All Aβ+ = all amyloid-β-positive participants (CU Aβ+ and CI Aβ+ combined); ERC = entorhinal cortex; BA35/36 = Brodmann area 35/36; PHC = parahippocampal cortex; AH = anterior hippocampus; PH = posterior hippocampus; WM = white matter.*

1. **CU Aβ−**

**
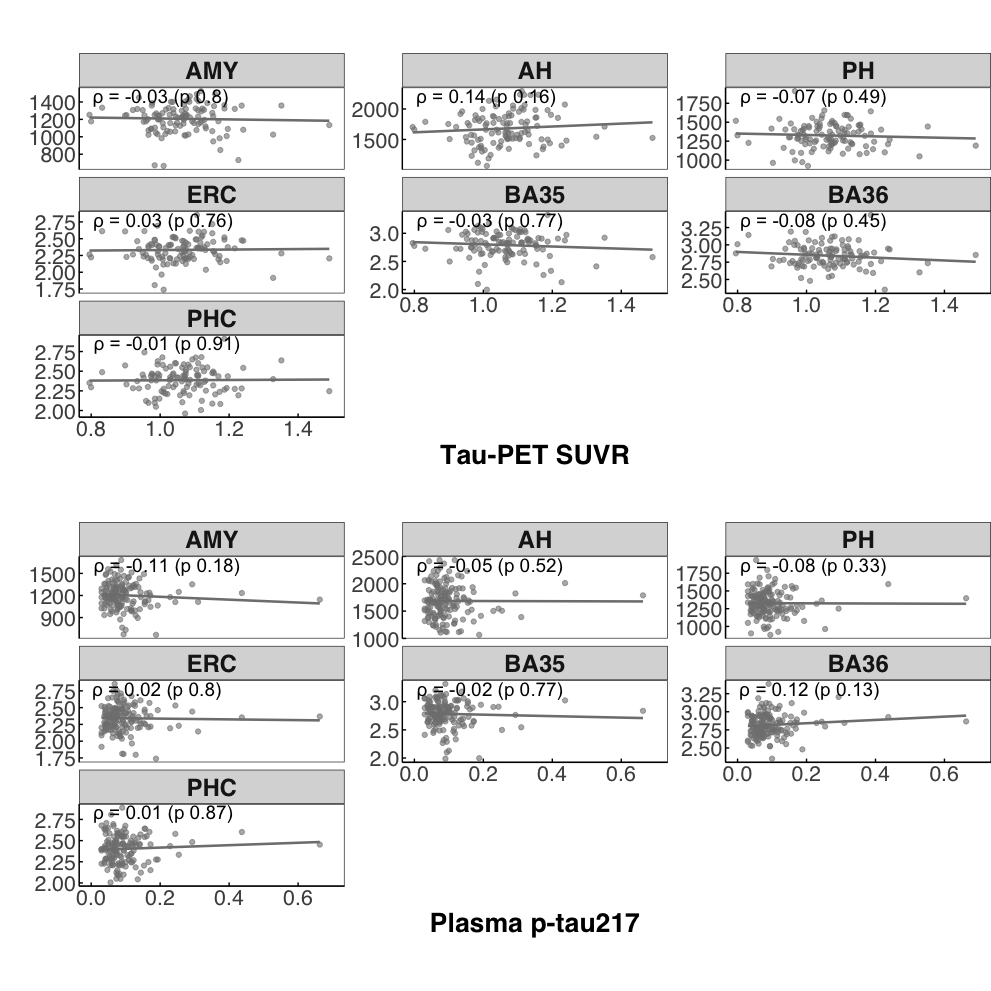
**

1. **CU Aβ+**
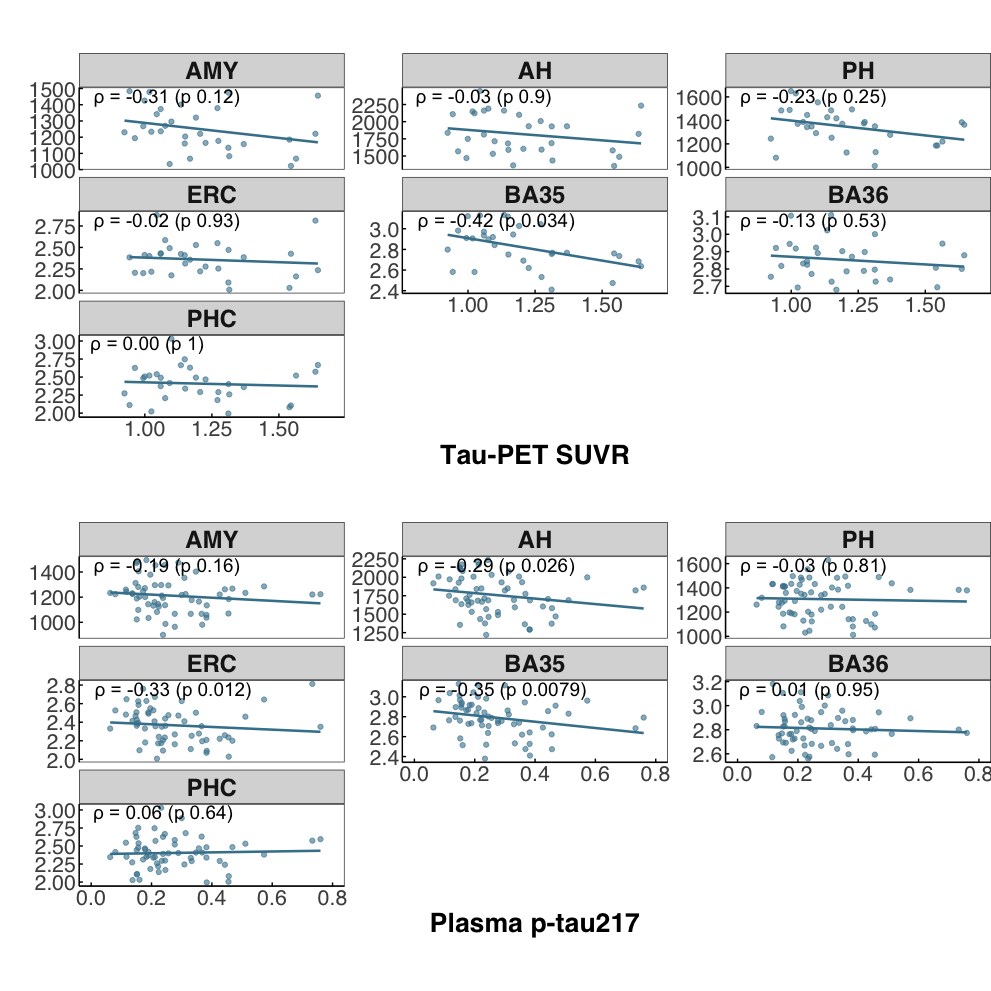

2. **CI Aβ+**

**
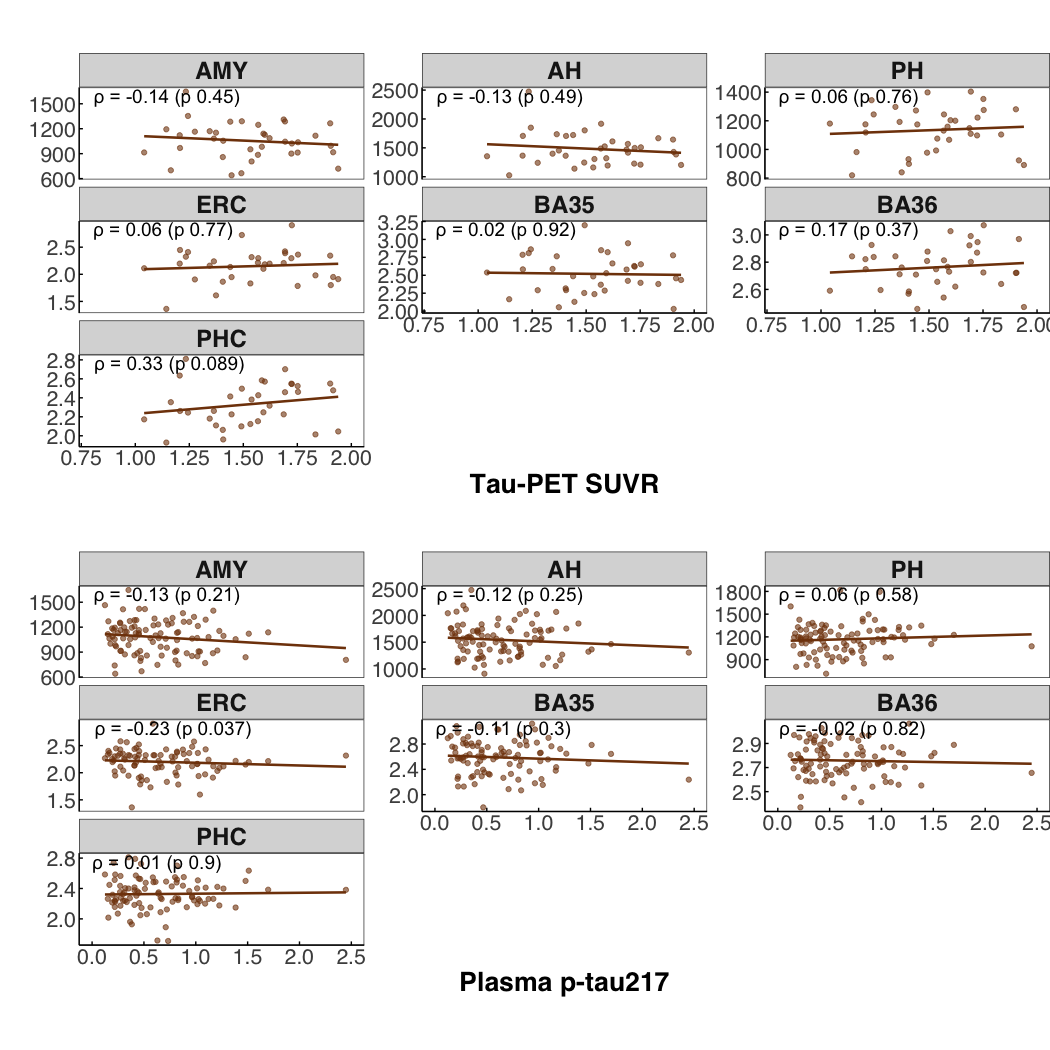
**

1. **All Aβ+**

**
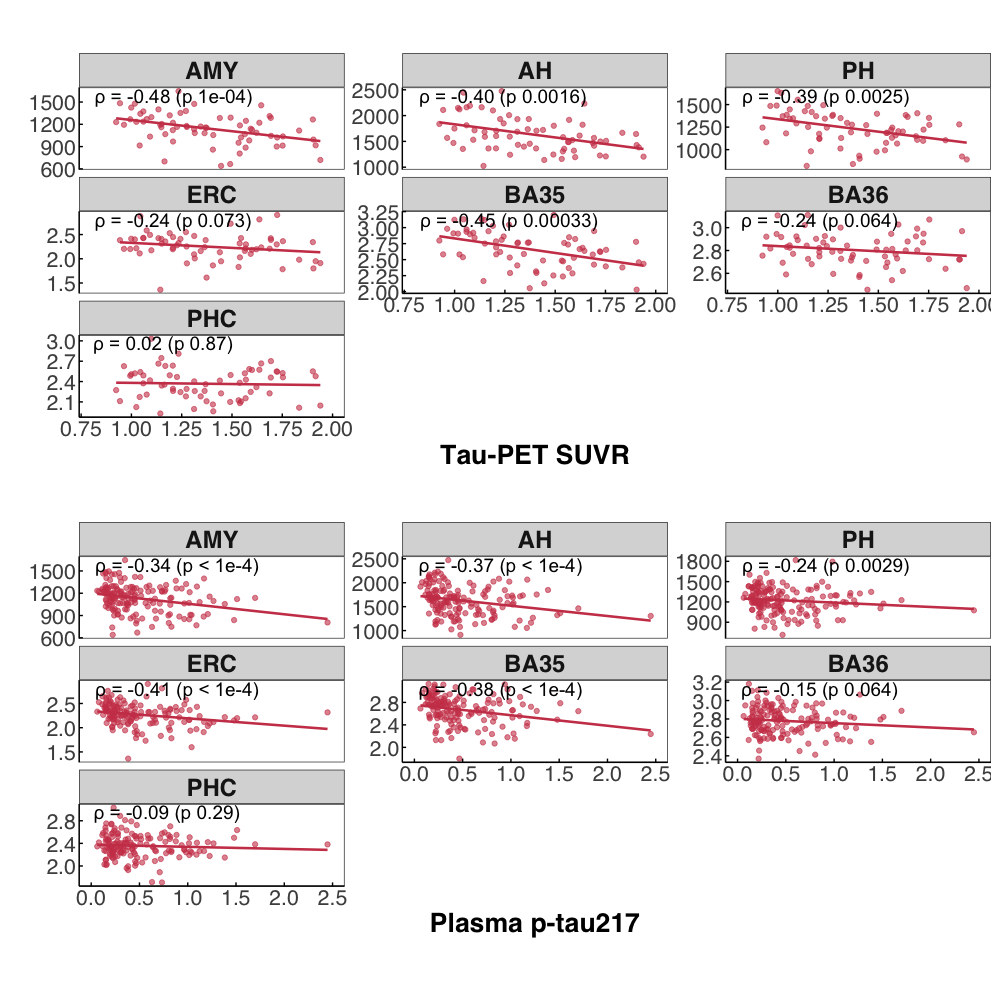
**

**Supplementary Figure 4. Scatterplot between tau biomarkers and MTL ROIs across groups**

*Partial Spearman’s correlations (ρ), controlling for age, sex, and ICV, reported for each correlation. (A) CU Aβ−= cognitively unimpaired amyloid-β-negative; (B) CU Aβ+ = cognitively unimpaired amyloid-β-positive; (C) CI Aβ+ = cognitively impaired amyloid-β-positive participants; (D) All Aβ+ = all amyloid-β-positive participants (CU Aβ+ and CI Aβ+ combined)*

**
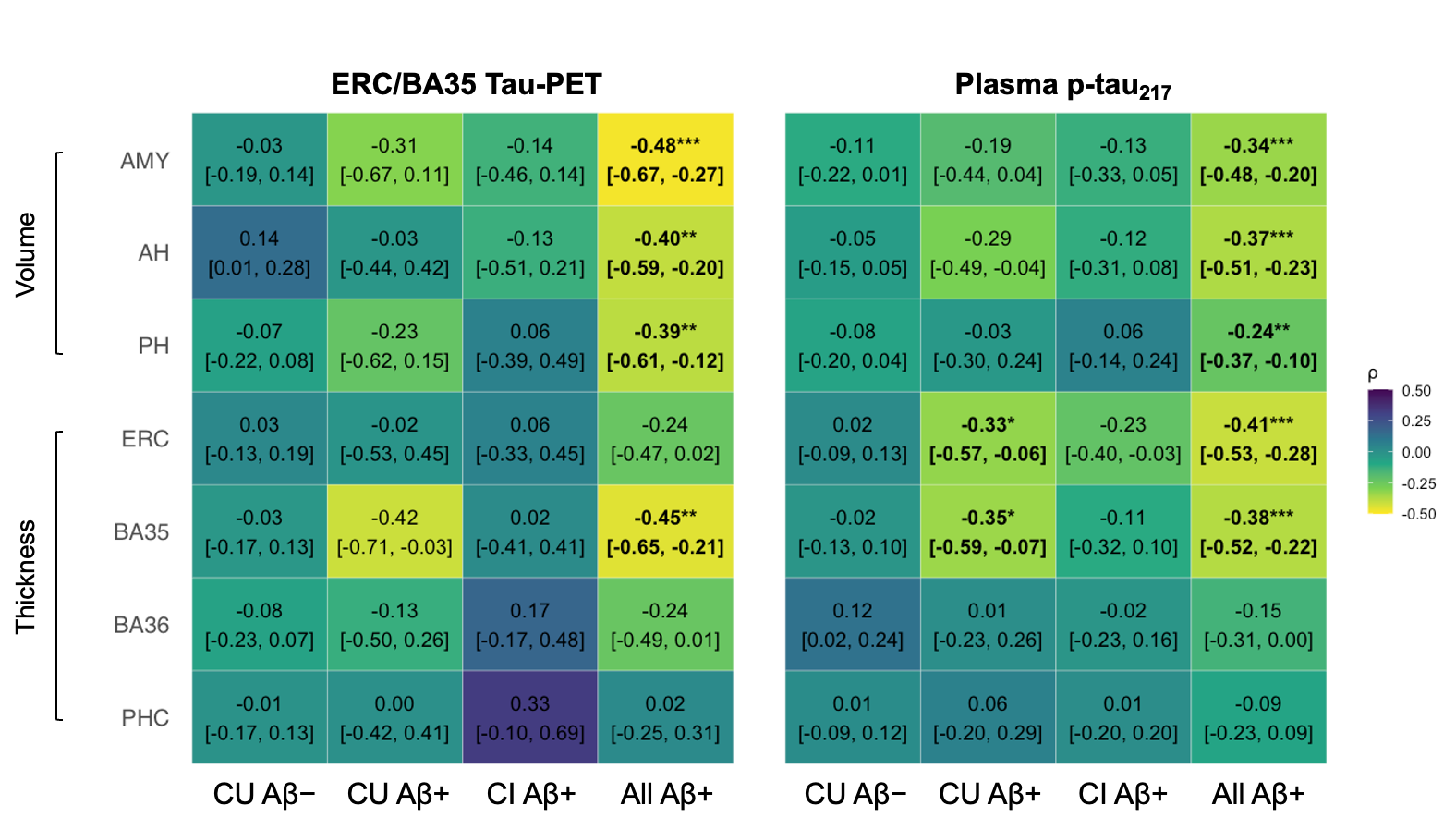
**

**Supplementary Figure 5. ROI-wise correlations with tau biomarkers after FDR-correction**

*Heatmaps of partial Spearman’s correlations (ρ), controlling for age, sex, and ICV, between regional MTL measures (amygdala [AMY] anterior hippocampus [AH], posterior hippocampus [PH], entorhinal cortex [ERC], Brodmann Area 35 [BA35], BA36, parahippocampal cortex [PHC]) and tau biomarkers. Correlation coefficients are shown with 95% confidence intervals estimated using bootstrap resampling (R=1000). Results are shown for tau-PET (left) and plasma p-tau_217_ (right), stratified by CU Aβ−, CU Aβ+, CI Aβ+, and All Aβ+ groups. Values inside tiles show correlation coefficients (ρ) (bolded when significant at *p < 0.05, **p < 0.01, ***p < 0.001), p-values are false discovery rate (FDR)–corrected. A consistent pattern of stronger associations in BA35, ERC, and hippocampal subregions is observed, with plasma p-tau_217_ showing robust effects across groups.*


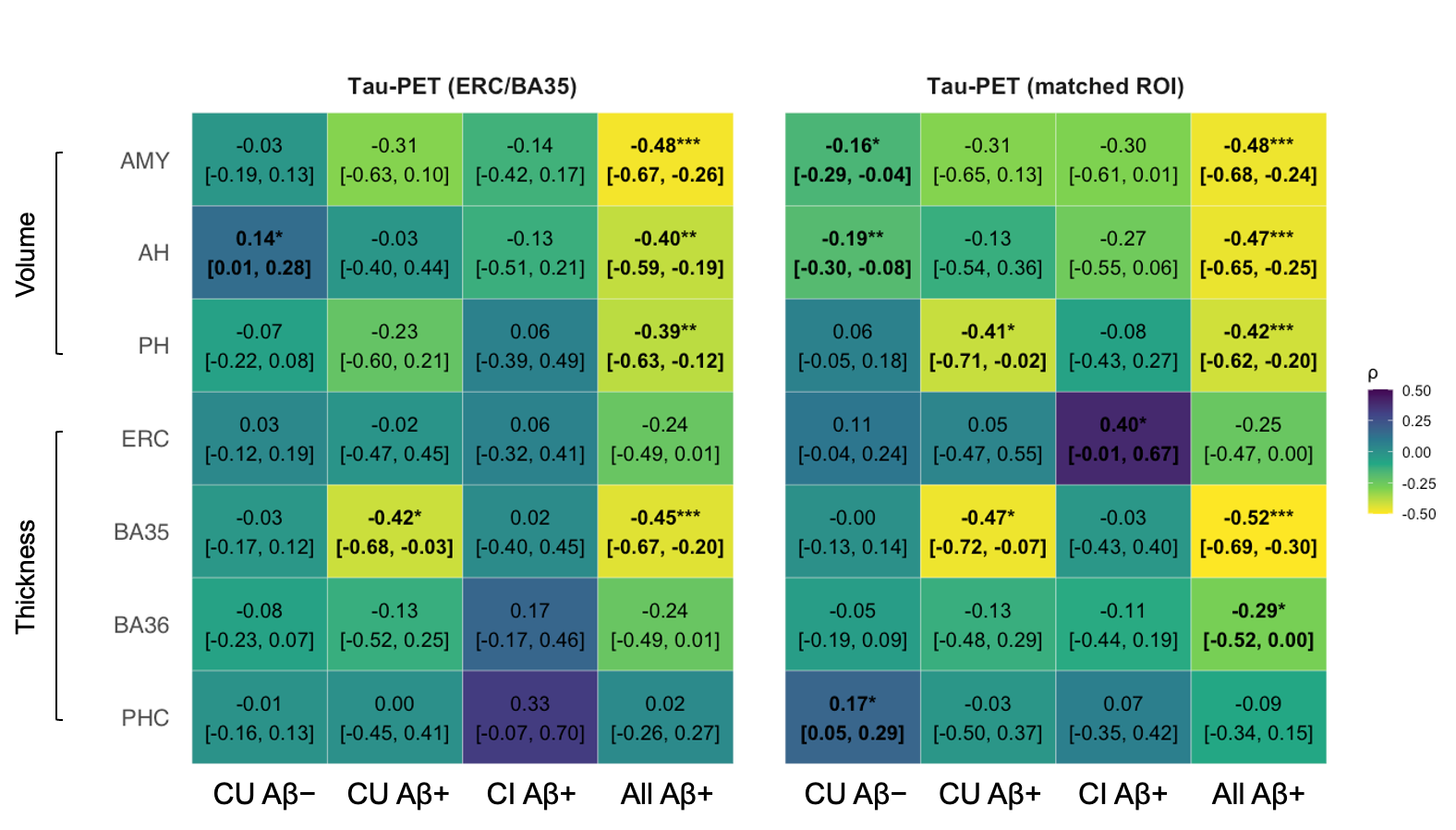


**Supplementary Figure 6. ROI-wise correlations with tau-PET (ERC/BA35 SUVR versus matched-ROI SUVR) without FDR-correction including CI Aβ+ group separately**

*Heatmaps of partial Spearman’s correlations (ρ), controlling for age, sex, and ICV between regional MTL measures (amygdala [AMY] anterior hippocampus [AH], posterior hippocampus [PH], entorhinal cortex [ERC], Brodmann Area 35 [BA35], BA36, parahippocampal cortex [PHC]) and ERC/BA35 SUVR (left) or matched-ROI SUVR (right). Mean choroid-plexus tau-PET SUVR was also included as a covariate for matched ROI analyses to account for off-target binding. Correlation coefficients are shown with 95% confidence intervals estimated using bootstrap resampling (R=1000). Results are shown for matched ROI tau-PET SUVR (left) and plasma p-tau_217_ (right), stratified by CU Aβ−, CU Aβ+, CI Aβ+, and All Aβ+ groups. Values inside tiles show correlation coefficients (ρ) (bolded when significant at *p < 0.05, **p < 0.01, ***p < 0.001). A consistent pattern of stronger associations in BA35, ERC, and hippocampal subregions is observed, with plasma p-tau_217_ showing robust effects across groups.*

**
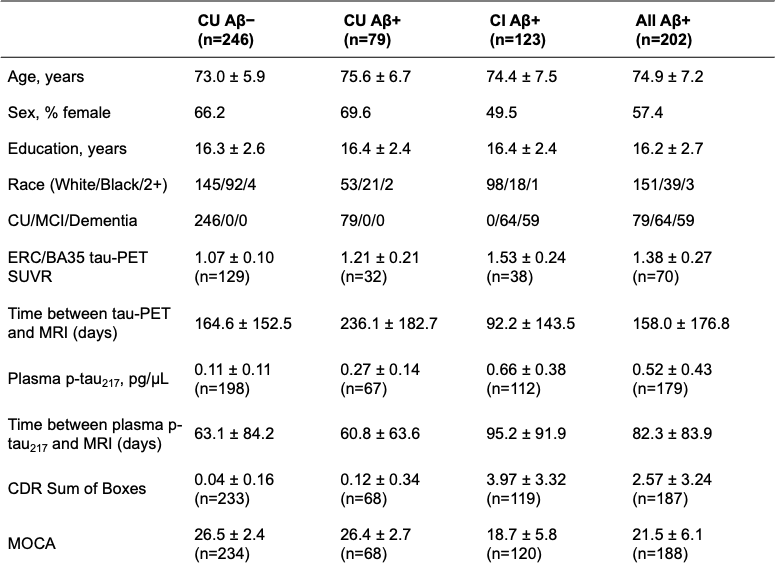
**

**Supplementary Table 1. Participant characteristics by group including CI Aβ+ group separately**

*Values are presented as mean ± standard deviation (SD) for continuous measures and percent for categorical variables. Sex is shown as % female. Race shown as counts for White, Black or African American, and more than 2 races. For biomarker measures, the number of participants with available data is indicated in parentheses. CU Aβ−= cognitively unimpaired amyloid-β-negative; CU Aβ+ = cognitively unimpaired amyloid-β-positive; CI Aβ+ = cognitively impaired amyloid-β-positive; All Aβ+ = all amyloid-β-positive participants (CU Aβ+ and CI Aβ+ combined); ERC = entorhinal cortex; BA35 = Brodmann area 35; SUVR = standardized uptake value ratio.*

**
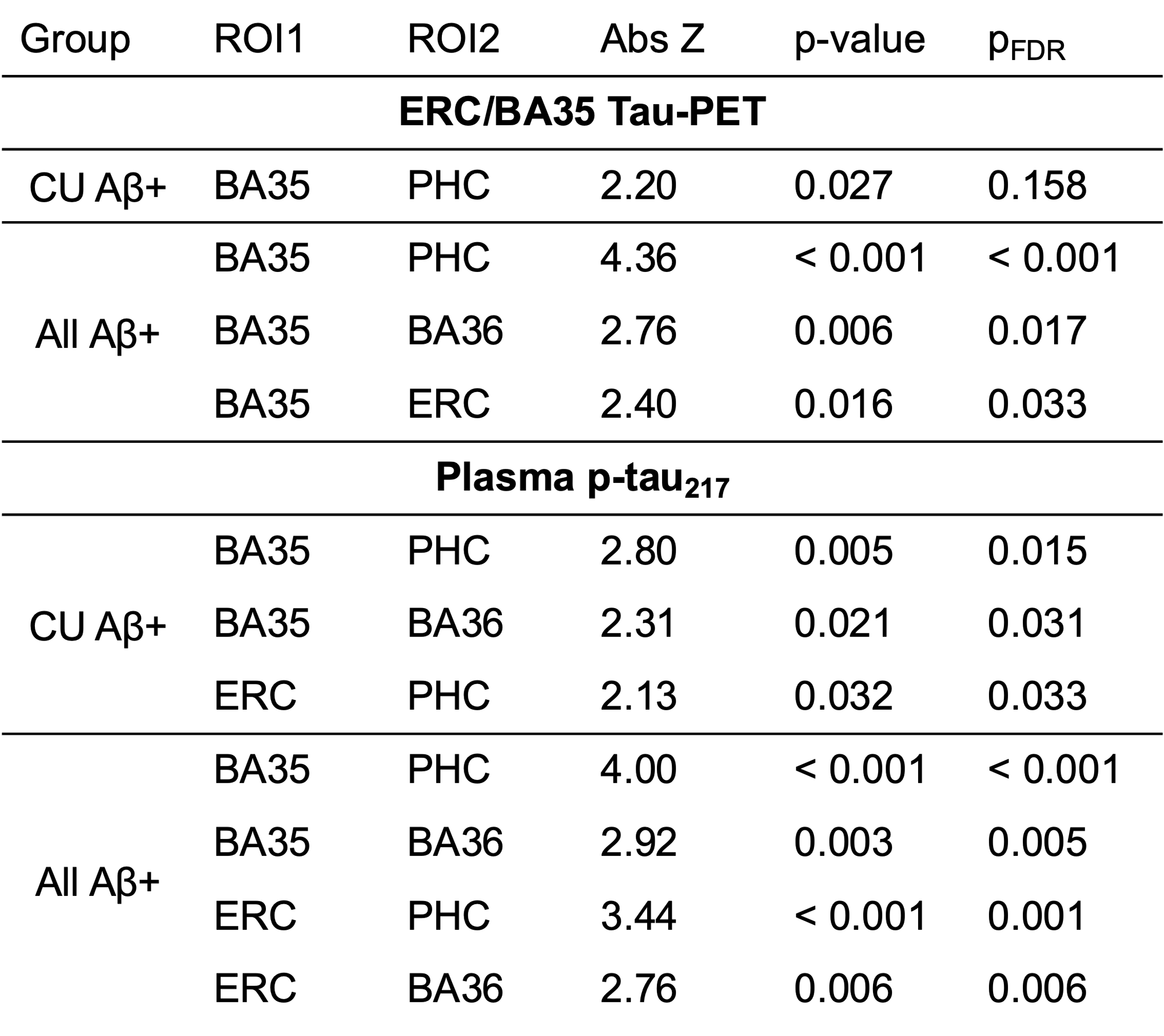
**

**Supplementary Table 2. Hittner’s Z tests comparing the strength of correlations between tau biomarkers and regional MTL measures**

*Tests were conducted only for ROI pairs in which at least one region showed a significant association with the corresponding tau biomarker. Positive Z-values indicate stronger correlations for ROI 1 relative to ROI 2. Only significant results (p < 0.05) are shown.*
